# Current Researches on Nanodrug Delivery Systems in Bladder Cancer Intravesical Chemotherapy

**DOI:** 10.3389/fonc.2022.879828

**Published:** 2022-05-24

**Authors:** Yilei Lu, Siqi Wang, Yuhang Wang, Mingshan Li, Yili Liu, Dongwei Xue

**Affiliations:** Department of Urology, The Forth Hospital of China Medical University, Shenyang, China

**Keywords:** bladder cancer, intravesical chemotherapy, nano-drug delivery system, liposomes, polymers, inorganic material

## Abstract

Bladder cancer is one of the most common malignant tumors in urinary system. Intravesical chemotherapy is a common adjuvant therapy after transurethral resection of bladder tumors. However, it has several disadvantages such as low drug penetration rate, short residence time, unsustainable action and inability to release slowly, thus new drug delivery and new modalities in delivery carriers need to be continuously explored. Nano-drug delivery system is a novel way in treatment for bladder cancer that can increase the absorption rate and prolong the duration of drug, as well as sustain the action by controlling drug release. Currently, nano-drug delivery carriers mainly included liposomes, polymers, and inorganic materials. In this paper, we reveal current researches in nano-drug delivery system in bladder cancer intravesical chemotherapy by describing the applications and defects of liposomes, polymers and inorganic material nanocarriers, and provide a basis for the improvement of intravesical chemotherapy drugs in bladder cancer.

## Introduction

**Figure 1:**
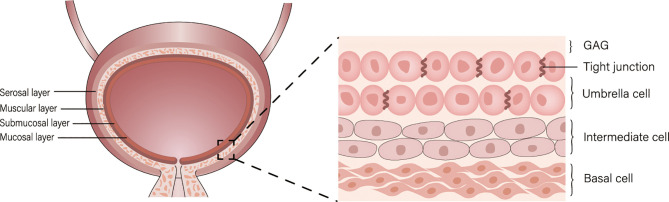
Levels of the bladder and bladder permeability barrier (BPB).

Bladder cancer (BC) is a common disease of the urinary tract, and its incidence ranks tenth in the world among oncological diseases (1). Approximately 75% of patients with BC present with disease confined to the mucosa (Ta or CIS) or submucosa (T1) (2), transurethral resection of bladder tumor (TURBt) is a common treatment for bladder cancer and often following intravesical chemotherapy (3). However, the bladder permeability barrier (BPB) including umbrella cells and glycosaminoglycans (GAG) on its surface affect drug penetration, and the regular emptying of the bladder dilutes or excretes the drug, resulting in a short drug residence time, which makes intravesical chemotherapy less efficient (**Figure 1**) (4). Therefore, new drug delivery systems are urgently needed to be developed.

In the field of medicine and pharmacology, nanoparticles are substances with diameters of 1-200 nm and 1-1000 nm separately. Nanomaterials are currently used to construct drug delivery carriers as a novel drug delivery system. Liposomes, polymers, and inorganic nanomaterials are the most dominant drug delivery carriers currently. These carriers exert longer duration of action and reduce side effects by controlling drug release, and can encapsulate multiple drugs to achieve combination therapy, which helps to improve the efficacy of intravesical chemotherapy (5–7). To provide a basis for the improvement of bladder cancer intravesical chemotherapy, we elaborate current researches on the application of nano-drug delivery systems. (**Table 1**)

**Table 1:** Summary of nano drug carrier.

Category	Subcategory	Material	Size	Carried Drug	Superiority	Reference
Liposome		Vesicles composed of phospholipid bilayers	100-500nm	Bacillus Calmette-Guerin Vaccine, Rapamycin	Carrying hydrophilic and hydrophobic drugs	(8–12)
Polymer	Gel	Gel	65-103nm	10-hydroxycamptothe- -cin, Adriamycin	Selective release, High adhesion and penetration, No obstruction of the urethra	(13–16)
	Chitosan	Chitosan	<150nm	Mitomycin C, Adriamycin, Nitazoxanide	Enhance drug penetration and adhesion into urothelium	(17–23)
	Microemulsions	Microemulsions	<100nm	Gemcitabine, Cisplatin	Thermodynamically stable and isotropic	(24–26)
	Micelles	Amphiphilic copolymer	15-80nm	Doxorubicin	Core-shell structure	(27–29)
Inorganic material nanocarrier	Mesoporous silica nanoparticles	Silica	50-200nm	Doxorubicin	large pore volume and specific surface area	(30–33)
	Metal nanoparticles	Fe_3_O_4_	5-10nm	Gemcitabine, Epirubicin, Methotrexate	Superparamagnetic, Nanochemothermia	(34–39)
Other	Nanomotor	Urease			Self-Promotion	(40, 41)

## Liposomes

**Figure 2:**
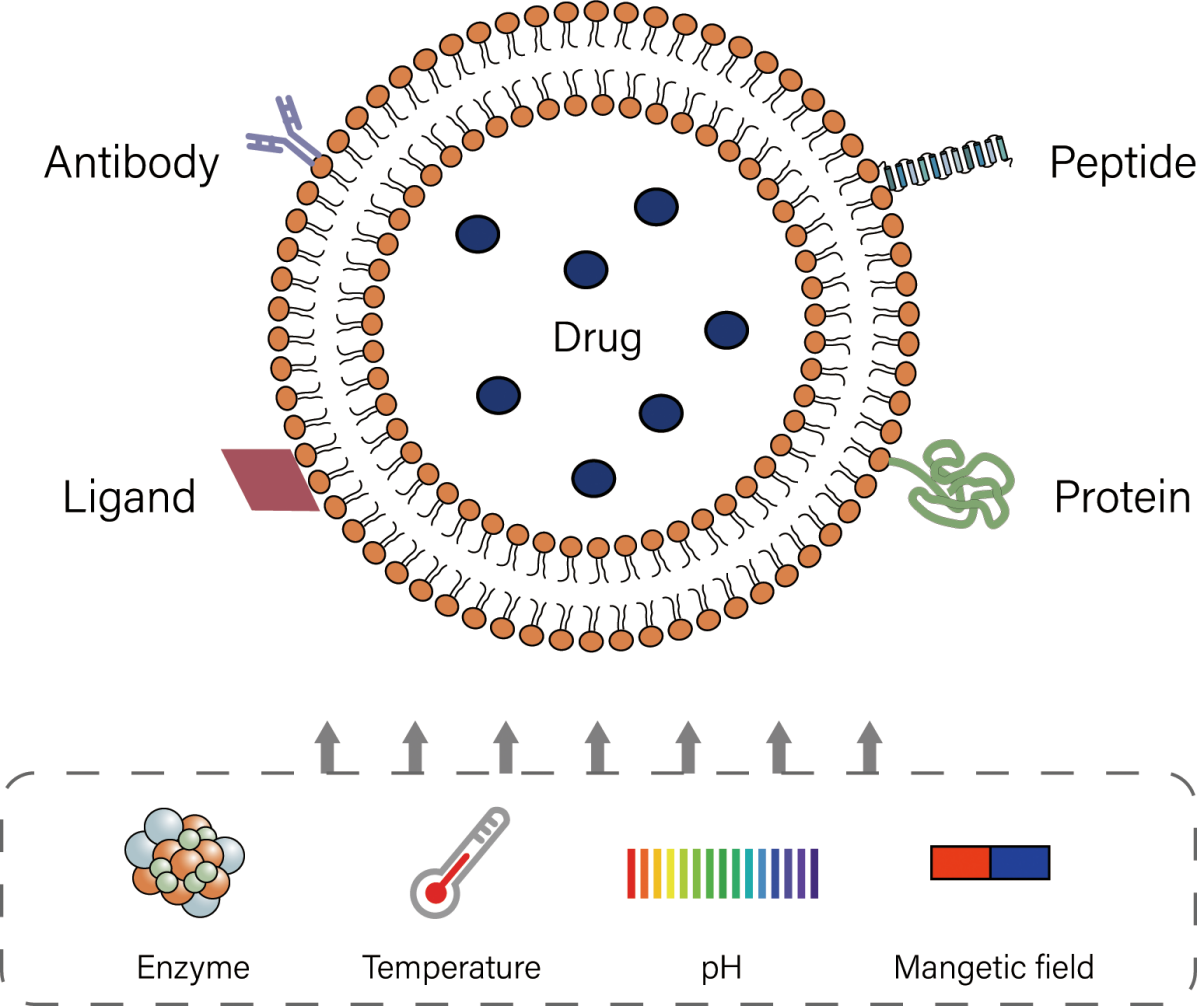
Modification of liposomes and various trigger release conditions of liposomes.

Liposomes are lipid based on spherical shaped vesicular systems, in which a lipophilic bilayer is sandwiched between two hydrophilic layers (8, 9). The biodegradability, biocompatibility and high encapsulation rate of liposomes, as well as their relatively simple production and stable properties, are of great significance in the clinical application of drug delivery (42, 43). During drug delivery, dynamic changes in the *in vivo* microenvironment of liposomes can promote the release of drug at specific locations or control the release in targeted tissues, which is known as “triggered release” (44). Liposomes mainly include thermosensitive, pH-sensitive, ultrasound-sensitive, enzyme-triggered, magnetic field-sensitive and ligand-targeted liposomes (45). Most liposomes currently used in infusion chemotherapy for bladder cancer are ligand-targeted (**Figure 2**). Though modification, liposomes show excellent effect in intravesical chemotherapy.

Modified liposomes can effectively overcome the poor water solubility of some drugs, which affects the cellular uptake rate and perfusion effect (46). Bacillus Calmette-Guérin (BCG) is an important agent for intravesical chemotherapy in non-muscle-invasive bladder cancer, but it has greater local or systemic adverse effects (47). BCG cell wall skeleton (BCG-CWS) has relatively few adverse effects and can replace live BCG for bladder perfusion, but it is poor in water solubility and low in cancer cells uptake (10). Nakamura et al. (10) obtained homogeneous and water-soluble nanoparticles (CWS-NP/LEEL) by the liposome evaporated *via* emulsified lipid (LEEL) method. CWS-NP/LEEL has a high uptake rate and significant tumor suppression ability in rats. Although the drug-loaded liposome preparation was enhanced by the addition of stearylated octaarginine (STR-R8) to enhance internalization, the problem of targeted drug uptake by cancer cells was not addressed.

Taking advantage of the high metabolism of tumors, modification of liposomes by specific metabolites can improve the targeted uptake of drugs by tumors. Folic acid (FA) is essential for tumor cell growth, and it has the advantages of high receptor affinity, small size, economy, and stability. In addition, folate receptor (FR) is deficiently expressed in normal cells and abundantly expressed on a variety of tumor cancer cells (48–52). Consequently, Yoon et al. (11) used folic acid (FA) and the cell-penetrating peptide (Pep1) to improve the encapsulation of LEEL and changed the solvent from pentane to dichloromethane to form a novel vector CWS-FPL (FA- and Pep1-modified liposomes). FA contributes to targeted drug delivery to cancer cells, and positively charged Pep1 facilitates drug delivery into the cells. The mean fluorescence intensity (MFI) of CWS-FPL uptake rates in both 5637 and MBT2 cell lines was significantly higher (138.26 and 132.59) than that of ordinary liposomes (34.95 and 35.2). CWS-FPL also significantly inhibited tumor growth. Rapamycin (Rap) encapsulated in FA-modified liposomes (R-FL) can effectively remedy its disadvantage of poor water solubility and improve the tumor killing ability. Co-cultured with low doses of Rap, plain encapsulated liposomes (R-CL) or R-FL for 48 h, the bladder cancer cell viability varied, Rap and R-CL decreased the viability less than 10%, while R-FL decreased the viability by 40%, demonstrating that R-FL performed a significant cytotoxic effect. This tumor inhibition ability resulted from the increased cell adhesion by FA modification (12).

Functionalized liposome also improves the poor retention and poor permeation of the drug in regular urination of the bladder (53). Kaldybekov et al. (54) explored maleimide-functionalized PEGylated liposomes (PEG-Mal) with efficient mucosal adhesion and penetration ability. PEG-Mal WO_50_ (wash out_50_, volume of artificial urine required to wash out 50% of liquid formulation) (55) was significantly higher than that of conventional liposomes (48 ml versus 15 ml), reflecting the excellence *in vitro* retention, fluorescence microscopy of PEG-Mal and conventional liposomes in the porcine bladder mucosa also showed good mucosal penetration, the release time of PEG-Mal was up to 8 h, which is significantly higher than the 2h saturation release time of conventional liposomes. This method effectively solves the problem of drug dilution and rapid loss due to urination.

## Polymers

**Figure 3:**
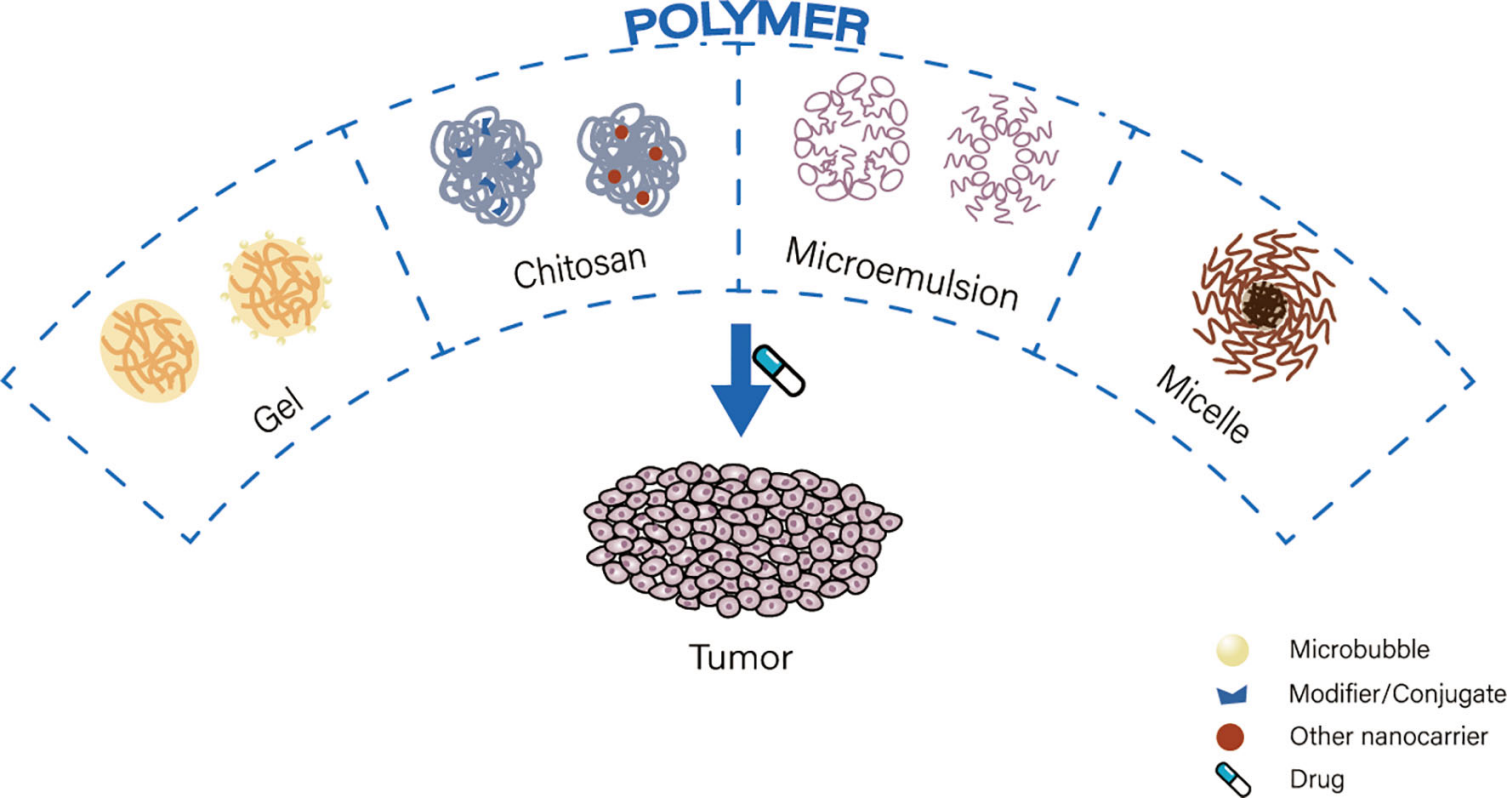
Classification of polymer nanoparticles.

Polymers are macromolecules composed of repeating subunits (56). Drug delivery carriers composed of polymers are characterized by controlled release times, biocompatibility, and hydrophilic and hydrophobic selective release (57). Polymeric carriers commonly used for nano-drug delivery are gels, chitosan, microemulsions and micelles (**Figure 3**).

### Gels

Gels are a hydrophilic three-dimensional polymer network (58) and are classified as macrogels, microgels and nanogels according to their size (59). Gels have sufficient adhesion to the uroepithelial mucosa and remain attached to the bladder wall after urination, which can avoid repeated drug instillation (60, 61). Nanogel delivery system can achieve floating or smart release, slow drug release and reduce lumen obstruction, this delivery system is appliable in intravesical chemotherapy (62–66).

The modified gel nanomaterials provide intelligent drug release and enhanced drug adhesion and penetration. Guo et al. synthesized a positively charged disulfide-core-crosslinked polypeptide nanogel of poly(L-lysine)–poly(L-phenylalanine-co-L-cystine) (PLL-P(LP-co-LC)) to form a drug-loaded nanogel (NG/HCPT) based on 10-Hydroxycamptothecin (HCPT). The release of NG/HCPT was slow in normal tissues and accelerated in tumor sites. Confocal laser scanning microscopy (CLSM) of whole bladder wall sections showed that the optical density of NG/HCPT at 0.5h, 2h and 6h was 1.7 times, 2.5 times and 5.3 times higher than that of free HCPT, reflecting good permeability. This carrier maintained a high concentration in bladder tissue and could penetrate the bladder wall, and the anti-tumor effect was obvious in animal model (13). In response to reduced efficacy in some patients after repeated treatments, Guo et al. also synthesized a smart disulfide-crosslinked polypeptide nanogel of poly-(l-lysine)-poly(l-phenylalanine-co-l-cystine), which triggered the breakage of the disulfide bond by high intracellular concentration of glutathione, and led to the selective release of hydroxycamptothecin from NG/HCPT (14). Then Guo et al. synthesized a new optimized oligoarginine-poly(ethylene glycol)-poly(L-phenylalanine-L-cysteine) nanogels (R9-PEG-P(LP-co-LC)). The PEG significantly improved the dispersion of the particles in water, and the non-specific interaction of PEG chains with bladder mucosa and the electrostatic interaction between cationic R9 and negatively charged bladder mucosa further enhanced the adhesion of the gels. Besides, as a cell-penetrating peptide, R9 effectively penetrated cell membranes and delivered drugs with more optimized antitumor effects than previous nanogels (67).

A floating gel modified by chemical reaction can compensate for ordinary gels defects that dislodged gel has direct contact irritation to bladder mucosa and can obstruct the urethra (68). A floating gel consisting of adriamycin (ADR), poloxamer 407 (P407) and NaHCO_3_ was developed, in which NaHCO_3_ produces microbubbles to float the gel in an acidic environment. This nanogel can avoid the possible direct contact and obstruction of the urinary tract (15). In addition, NH_4_HCO_3_ instead of NaHCO_3_ in modified floating gel can achieve temperature-controlled release. The microbubbles can be produced spontaneously at body temperature (37°C) and the gel float. This process was confirmed by ultrasound in rabbit bladder (16).

### Chitosan

Chitosan (CS) nanoparticles are applicable in drug delivery, transport, targeted drug uptake due to their excellent bioadhesion and permeability, unique polycationic, non-toxic and bioresorbable properties (69, 70). Similar to gel systems, chitosan plays an important role in bladder cancer perfusion therapy by modification through different chemical reactions or in combination with specific bioactive nanomaterials (71–76).

By coupling different substances, chitosan shows significant advantages in increasing the adhesion rate of perfused drugs. Some researchers have pioneered the reaction of chitosan with methacrylic anhydride to prepare methacrylate chitosan, and detected the adhesion and cytotoxicity on isolated porcine bladder. This methacrylate chitosan had a best adhesion effect compared with dextran and chitosan, and the adhesion ability depended on the methylation degree (17). Kolawole et al. (18) prepared high molecular weight chitosan (HCHI) with β-glycerophosphate to form an new nanocarrier, which was transparent and non-coagulable at room temperature. This nanocarrier binds to mitomycin C and forms a mucoadhesive gel layer with a large bladder area at elevated temperatures, allowing prolonged drug diffusion. However, this study was performed in isolated bladders filled with artificial urine, the specific adhesion, release efficiency and other potential defects need to be further verified and explored. In addition, the synthetic borate-coupled chitosan derivatives (FS/LBCHI) exhibited significantly elevated mucoadhesive properties by applying coupling agents to interact chitosan with 4-carboxyphenylboronic acid. It can be seen that coupling with modified chitosan significantly improves the adhesion of perfused drugs (19).Moustafa et al. used tripolyphosphate (TPP)-treated chitosan and loaded with nanodiamond-bound doxorubicin (DOX), which showed superior cytotoxic effects on human bladder cancer cells than DOX or NDs alone and increased drug retention in the isolated bovine bladder wall, suggesting that this carrier may enhance the drug effect of bladder perfusion (20, 21).

Besides, specific chitosan nanocarriers can increase chemotherapeutic drug bioavailability and enhance tumor suppression. Tumor-selective photosensitizer dyes in photodynamic therapy are retained and accumulated by aberrant or overproliferating cells (e.g., tumor cells) and combined with tissue oxygen and targeted illumination, intracellularly produce cytotoxic reactive oxygen species (ROS) to kill tumor cells and selectively destroy tissue in the diseased area (77, 78). Specific chitosan nanocarriers based on photodynamic therapy can increase the effect of chemotherapeutic agents. Nitazoxanide (NTZ) and chlorine e6 (Ce6)-conjugated human serum albumin (HSA-Ce6) formed self-assembled human serum albumin-Ce6/NTZ nanoparticles (NPs), which were further compounded across the mucosal carrier fluorinated chitosan (FCS) to form HSA- Ce6/NTZ/FCS nanoparticle. The highest AMPK α phosphorylation levels were detected in cells treated with this nanoparticle, which effectively improved tumor tissue hypoxia and inhibited tumor cell overproliferation. The 5-week survival rate of mice with *in situ* bladder cancer perfused with this nanoparticle reached 83%, which was significantly prolonged compared to 17%-33% in other treatment groups, and could effectively alleviate the effect of tumor hypoxia on drug resistance (22). Manan et al. developed a novel Mn : ZnS quantum dot-bound chitosan nanocarrier (CS-Mn : ZnS), which can load drug such as Mitomycin C (MMC) and promote engulfment of drug into target tumor cells efficiently and continuously. This chitosan nanocarrier improves the bioavailability of drugs, and it is possible to be a practical tool for intravesical perfusion chemotherapy (23).

### Microemulsions

Microemulsions are spontaneously formed single-phase dispersion systems, which are thermodynamically stable and isotropic. The small and uniform particle size of microemulsion can improve the dispersion of the drug sealed in it and promote the transdermal absorption of the drug (24, 25). Chen et al. (26) used a microemulsion carrier to carry gemcitabine and cisplatin. This carrier was chemically and physically stable, with significantly better permeability than the corresponding aqueous solution and significantly less bladder irritation than the chemotherapeutic drug alone.

### Micelles

Micelles are colloidal systems formed spontaneously by amphiphilic copolymers in aqueous media (27). In aqueous environment, hydrophobic chain segments gather each other to form a hydrophobic core due to the exclusion of water molecules, while hydrophilic chains surround them to form a hydrophilic layer, eventually forming micelles with a core-shell structure that remains stable in water. Unlike liposomes, micelles have only one lipid layer, ranging in size from 15 nm to 80 nm (28). DOX and IR780 dyes (a near infrared dye) were used to form self-assembled micelles (DOX&IR780@PEG-PCL-SS NPs) with PEG-PCL-SS (An amphiphilic copolymer containing disulfide bonds) and cross-linked them internally to form nanoparticles under disulfide bonding (DTT) catalytic conditions. The DOX release rate was faster in bladder cancer cells with high GSH concentration. In addition, the photothermal effect of the nanoparticles by the photosensitizer IR780 could also greatly promote the release of the drug. The good photothermal properties and tumor targeting of this micelle gives it a greater advantage in bladder intravesical chemotherapy (29).

## Inorganic Material Nanocarriers

Common inorganic drug nanocarriers can be broadly classified into non-metallic nanoparticles and metallic nanoparticles, which show promising applications in targeted drug delivery, controlled release and sustained release of drugs (79).

### Non-metallic Nanoparticles

Mesoporous silica nanoparticles (MSNPs) are the more widely used carriers among the non-metallic nanoparticle types. Their excellent biocompatibility, high stability, rigid backbone, good pore structure, tunable surface chemistry and controlled release for drugs, determine they to be excellent drug carriers (30, 31). Wei et al. (32) prepared novel targeting adriamycin mesoporous silica nanoparticles and peptide CSNRDARRC couples (DOX-loaded MSNs@PDA-PEP), which could load DOX more efficiently compared to MSNs alone. It had higher internalization rate and targeting efficiency in HT-1376 cells, which could improve the therapeutic effect on bladder cancer and show non-toxicity to mice model. Another highly mucoadhesive nano-drug delivery system was prepared using poly-amidoamine (PAMAM) modified MSNPs (MSNPs-G0~MSNPs-G3) and loaded with DOX. With the dilution of urine, the release of DOX increased significantly with the decrease of pH, in addition MSNPs-G2 had the best mucoadhesive property and the mucoadhesive ability of MSNPs-G2 remained unchanged after loading DOX (33).

### Metal Nanoparticles

#### Common Metal Nanoparticles

Currently, metal nanoparticles are often prepared using biological systems, which are nontoxic, economical and highly efficient compared to traditional physical or chemically mediated methods, and their applications are gradually expanding to bladder cancer treatment (80). Ferreira et al. synthesized Fusarium biogenic silver nanoparticle for the treatment of NMIBC patients who are highly malignant, ineffective with BCG treatment or relapsed, this nanoparticle can directly induce DNA damage and have significant antitumor effects *in vivo* (81). Cuprous oxide nanoparticles (CONPs) could activate the ROS/ERK signaling pathway to induce apoptosis in bladder cancer cells, and they were more metabolizable, less toxic, and more suitable as drug carriers for intravesical chemotherapy. Combining CONPs with gemcitabine chemotherapy reduced the recurrence rate by synergistically exerting a more optimal effect than single agents (82). In addition, studies have also confirmed the killing effect of gold nanoparticles on different bladder cancer cell lines with the same effect (83, 84).

#### Magnetic Nanomaterials

**Figure 4:**
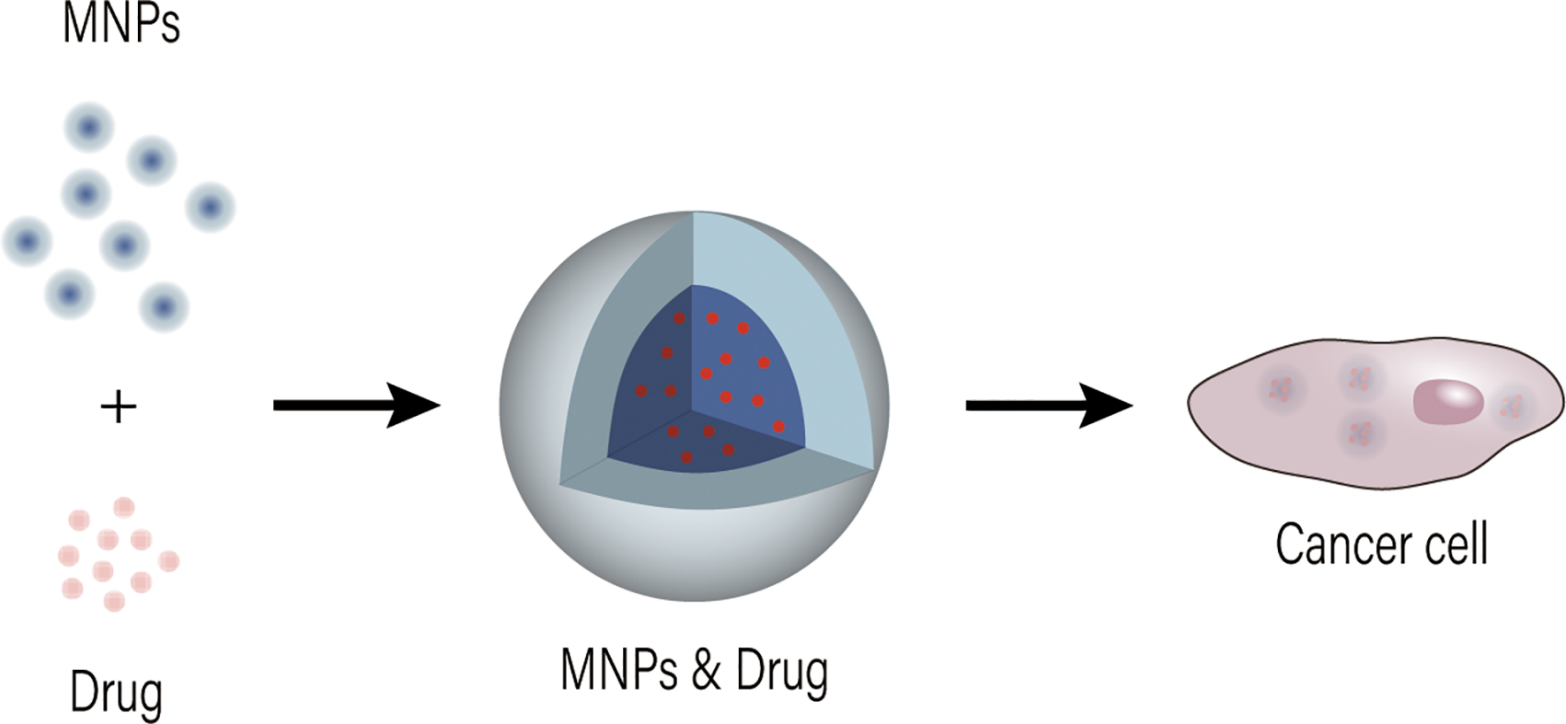
Drug delivery mode and action of MNPs on tumor cells.

Magnetic nanoparticles (MNPs) are dominated by oxides of iron. MNPs are appropriately sized, easily prepared and surface modified, with good adsorption capacity and they can combine with magnetic guidance (**Figure 4**). These characteristics make MNPs as important tools in drug delivery, imaging and clinical diagnosis (34, 35). With appropriately size, MNPs can increase endocytosis by cancer cells. Jasna Lojk et al. (85) synthesized polyacrylic acid (PAA)-coated MNPs and studied the differences in their endocytosis in normal primary urothelial (NPU) cells, RT4 cell line and T24 cell line. They confirmed that cancer cells can selectively take up this vector. The surface modification of MNPs can facilitate the binding of antitumor drugs and the targeted uptake by tumor cells. Suo et al. prepared a novel magnetic carboxylated multiwalled carbon nanotubes (mMWCNTs) for loading epirubicin (EPI). Application of such magnetic multi-walled carbon nanotubes not only alleviated the toxicity of the drug to normal cells, but also increased the dispersion and efficiency of the loaded drug, resulting in good stability of the solution and anti-tumor activity *in vitro* and *in vivo* (36). In addition, magnetic fields acting on MNPs can lead to an increase in local temperature and thus kill tumor cells (37, 38). Studies have shown that methotrexate coupled with MNPs (MTX/MNPs) in combination with magnetic heat achieves better cancer suppression than drug therapy alone and adjuvant heat therapy alone. The advantage of MTX/MNPs combined with magnetic heat therapy was the low CEM43T90 value (the cumulative equivalent minutes representing 43°C in 90% of the tumor area) was sufficient for rapid tumor destruction and no recurrence (39). Further the superparamagnetic nature of magnetic nanomicrospheres allows for tumor localization and vascular imaging for early diagnosis of disease (11, 32, 86, 87). These advantages of MNPs can reduce the toxicity of perfused drugs, induce slow and sustained drug release, increase targeted binding, uptake and targeted killing of tumor cells, and will enhance the efficacy of perfusion chemotherapy for bladder cancer.

## Others

In addition to the above mentioned nanocarriers, there are some special carriers that can be applied for chemotherapy of bladder cancer. With self-propulsive properties, nanomotors show great potential in overcoming drug delivery barriers (88). One investigator prepared urease powered nanomotors of MSNPs containing both polyethylene glycol and anti-FGFR3 antibodies on their outer surface. The urease driven nanomotor converts urea to carbon dioxide and ammonia thereby triggering the propulsion of the nanomotor. The targeting functions of substrate-dependent enzyme nanomotors was demonstrated in spheroid culture (3D culture) of human bladder cancer cells (40). Choi et al. prepared a biocompatible and bioavailable nanomotor using dopamine (PDA) hollow nanoparticles. This nanomotor was fluorescently labeled and showed strong fluorescence in the bladder wall even after 12 hours of perfusion and penetrated to a greater depth than the control group (41). Active motion increased the penetration ability of the nanomotor, and active antibody-modified nanomotors were more efficient than these without antibody modification.

## Application of Nanocarriers in the Diagnosis and Monitoring of Bladder Cancer

The diagnosis and follow-up of BC are mainly based on cystoscopy and urine cytology. Cystoscopy is invasive and costly, while urinary exfoliative cytology has low sensitivity (89). The efficient binding and easy detection characteristics of nanocarriers may become an important imaging tool for the diagnosis and monitoring of bladder cancer. One investigator used chitosan and ferromagnetic iron oxide nanocubes to design peptide-conjugated chitosan nanoparticles (pMCNP). The nanoparticles were administered to mice through the tail vein, and then good MRI and optical dual-modality imaging were detected. The pMCNP loaded with vincristine accumulated at the tumor site and showed controlled release for up to 50 hours (90). Besides the multi-binding site modification of MNPs has the potential for drug perfusion with simultaneous imaging detection (91). MNPs can also be used for urine protein capture and detection. Researchers synthesized a novel bladder cancer biosensor based on polycrystalline silicon nanowire field effect transistor (Poly-SiNW-FET) for the quantification of apolipoprotein A II protein (APOA2) in urine. The biosensor can clearly differentiate urine from non-bladder cancer patients, and the results are consistent with those of suspension chip analysis system (Bio-Plex). The detection is non-invasive, simple and fast. In addition, the biosensor accelerates purification during immobilization of anti-APOA2, effectively preventing denaturation of anti-APOA2 and increasing the accuracy of the assay (92). MNPs can selectively capture urinary glycoproteins from BC patients, contributing to the study of glycoproteome and having the potential to uncover glycoprotein biomarkers (93). Magnetic nanocarriers can also be used for the detection of exfoliated tumor cells. Xu et al. combined Fe_3_O_4_ and SiO_2_ to form new positively charged multifunctional nanoprobes that can specifically capture and enrich tumor cells in urine in a magnetic field, increasing the sensitivity of the detection (94).

## Discussion

The recurrence rate after TURBT for NMIBC is 50% to 80% (95), and prevention of bladder cancer recurrence after surgery is a key aspect to improve the prognosis of bladder cancer patients. Bladder perfusion chemotherapy is an effective mean of preventing tumor recurrence, but the tumor suppressive effect of the drugs reduces due to the barrier effect of bladder epithelium and regular urination behavior (61, 96, 97). Through the application of nanocarriers, both lipid-soluble drugs and water-soluble drugs can better act on tumor location. Meanwhile, drug adhesion, targeted uptake and killing to cancer cell increases, toxicity, release rate and adverse effects reduce. In addition, nanocarriers provide a low-invasive and efficient way to monitor bladder cancer through specific imaging and targeted binding detection of urine proteins or tumor cells.

However, there are still some drawbacks in the application of nanocarrier technology in bladder cancer intravesical chemotherapy. Firstly, liposome-based carriers have low water solubility, large molecular weight, non-uniform particle size, aggregate formation, and even affect the drug effect. Liposomes still have a certain amount of binding between carriers and healthy cells (28), which also proves that there may be toxic side effects on normal cells. In contrast, most chemically cross-linked hydrogel injections require high pressure and long delivery time when using small-diameter catheters for bladder perfusion (68), resulting a high attrition. Some polymer-based carriers are cumbersome to prepare, with complicated preparation processes and high development costs, limiting their translation to the clinic (36). Inorganic nanomaterial carriers are also in the early stage of research, lacking exact animal experiments and clinical experiments for effect verification. The targeting, drug resistance, encapsulation rate and release rate of the carriers themselves still need to be optimized and improved.

The high recurrence rate of bladder cancer, the limitations of perfusion drugs and their organ specificity require continuous improvement of perfusion drugs and drug delivery system, and the innovation and optimization of nano-delivery system is expected to provide a guiding idea for perfusion chemotherapy of bladder cancer and become a powerful tool for the treatment of bladder cancer.

## Author Contributions

DX contributed to design of the review. YLu and SW collected the data and information. LY wrote the first draft of the manuscript. YW wrote parts of the manuscript. ML and YLi embellished the manuscript. All authors participated in the revision of the manuscript and read and approved the submitted version.

## Conflict of Interest

The authors declare that the research was conducted in the absence of any commercial or financial relationships that could be construed as a potential conflict of interest.

## Publisher’s Note

All claims expressed in this article are solely those of the authors and do not necessarily represent those of their affiliated organizations, or those of the publisher, the editors and the reviewers. Any product that may be evaluated in this article, or claim that may be made by its manufacturer, is not guaranteed or endorsed by the publisher.
